# A Case of Nonuremic Calciphylaxis in a Caucasian Woman

**DOI:** 10.1155/2017/6831703

**Published:** 2017-01-16

**Authors:** Bonnie Fergie, Nishant Valecha, Andrew Miller

**Affiliations:** ^1^Canberra Hospital, ACT Health, Canberra, ACT, Australia; ^2^Woden Dermatology, Phillip, ACT, Australia

## Abstract

We report a case of nonuremic calcific arteriolopathy (NUCA) in an 82-year-old Caucasian woman from rural Australia. The patient had no history of kidney disease or dialysis. NUCA is rare disease suspected on cutaneous and clinical features and diagnosed by characteristic findings on skin biopsy and vasculature imaging. Calcification induced microvascular occlusion in the absence of renal failure may not be immediately apparent. Clinical suspicion and appropriate investigations are essential for making a diagnosis. A diagnosis of NUCA may be missed given the rarity of the disease, and dermatologists and patients alike would benefit from a greater awareness of this disease.

## 1. Introduction

Calcific uremic arteriolopathy (CUA), otherwise termed calciphylaxis, characteristically arises in the setting of end stage renal failure and hyperparathyroidism. Calcific arteriolopathy in the setting of normal renal function is a rare entity termed nonuremic calcific arteriolopathy (NUCA). Both NUCA and CUA are caused by microvascular occlusion secondary to calcium deposition in the vessels of the skin and subcutaneous fat. Given the high morbidity and mortality associated with NUCA, recognition and timely diagnosis are essential for patient management.

## 2. Case

An 82-year-old woman from rural Australia admitted to hospital for an exacerbation of congestive cardiac failure (CCF) developed intensely painful, flesh colored, subcutaneous nodules on both lower legs and left thigh on a background of livedo racemosa. The nodules coincided with a creatinine rise from 96 *μ*mol/L to 120 *μ*mol/L (Table 1) following aggressive diuresis with frusemide for CCF management. Frusemide was subsequently reduced and renal function normalized. Over the subsequent weeks the nodules ulcerated and took on a serpiginous form.

The patient's comorbidities were rheumatoid arthritis, atrial fibrillation, chronic obstructive pulmonary disease, mitral valve replacement (bovine), hypertension, and hypercholesterolemia. Her medications were frusemide, methotrexate 5 mg/week, prednisolone 2.5 mg/day, spironolactone, oxycodone, rabeprazole, potassium, warfarin, salbutamol, budesonide, and formoterol. She had been on the same dose of warfarin for many years and had never smoked cigarettes.

**Figure 1:**
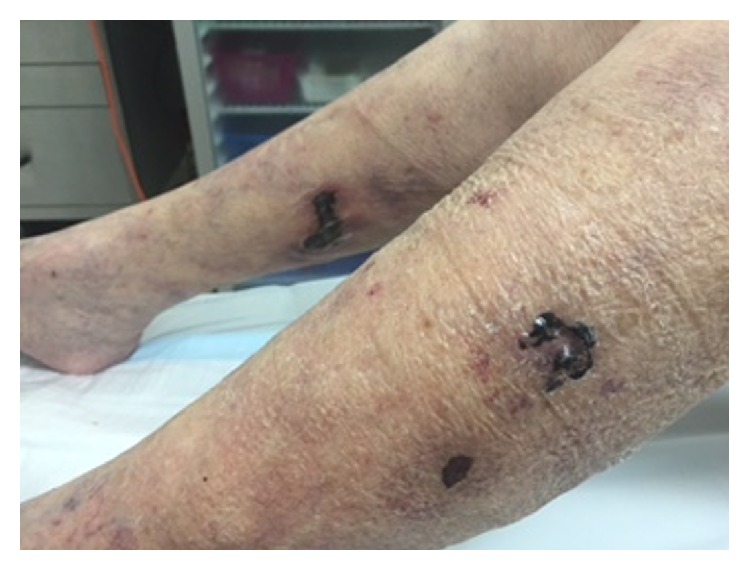
Necrotic ulceration on lower legs.

Physical examination showed an afebrile, haemodynamically stable, elderly woman in severe pain from the leg ulcerations. Multiple deep serpiginous ulcerations with overlying necrotic eschars and surrounding macular erythema were present on both lower legs (Figure 1). A new subcutaneous flesh colored nodule with surrounding erythema was present on her left lateral thigh. Livedo racemosa was present on her upper and lower legs bilaterally.

**Figure 2:**
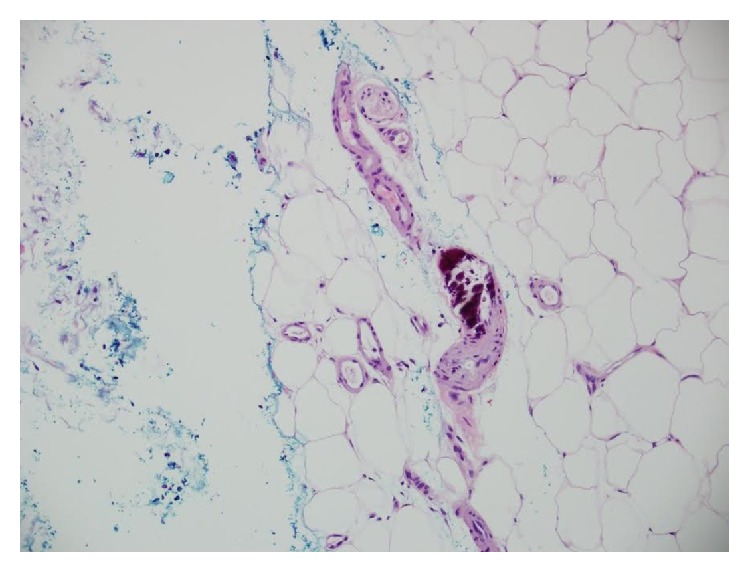
Biopsy left calf, illustrating arteriolar calcification in subcutis.

**Figure 3:**
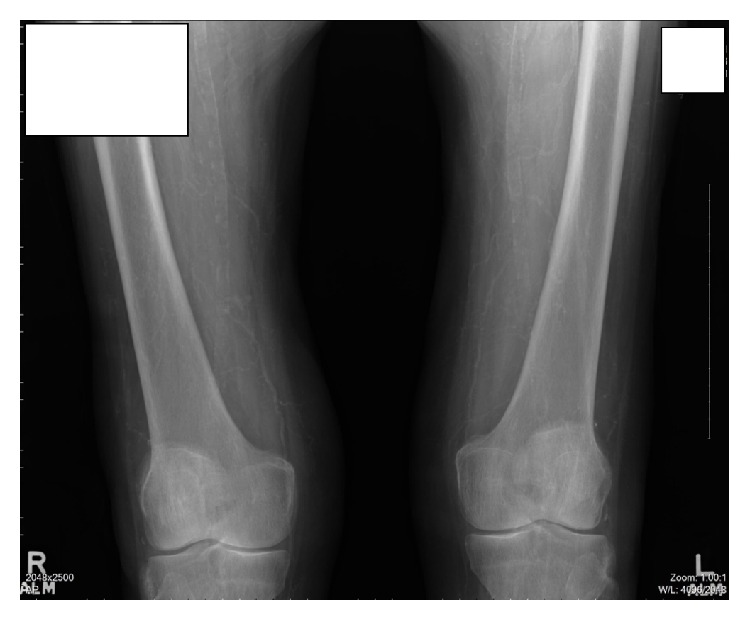
X-ray illustrating calcification of the femoral and popliteal vessels.

Deep wedge incisional biopsies taken from the left thigh nodule and calf showed panlobular infarction of subcutaneous fat with extensive mural calcification of small and medium sized vessels within the pannus, with luminal occlusion (Figure 2). The left calf showed epidermal and dermal necrosis and ulceration. No features of vasculitis were seen. Direct immunofluorescence was negative. Laboratory investigations (Table 1) showed an elevated intact parathyroid hormone (PTH) 17.7 pmol/L, low urine calcium 0.9 mmol/24 hr, and the aforementioned creatinine elevation. Serum calcium, phosphate, vitamin D, and urea were normal. Plain X-rays of the legs showed extensive vascular calcification bilaterally throughout the whole legs including the iliac, femoral, popliteal, and tibial vessels (Figure 3). Abdominal ultrasound showed areas of bilateral medullary nephrocalcinosis. Thyroid ultrasound showed a colloid cyst; the parathyroid glands were not visualized. Nuclear imaging of the parathyroid glands showed no abnormality. These clinical, radiological, and histopathological findings were consistent with a diagnosis of NUCA.

The patient was commenced on doxycycline 50 mg/day and was given an infusion of Zoledronic acid 5 mg. Warfarin was ceased and the patient was commenced on rivaroxaban. There were no new nodules 6 months after the initial episode and the established erosions were healing slowly.

## 3. Discussion

NUCA is a rare obstructive vasculopathy caused by calcium deposition within the lumen of small and medium sized blood vessels. In the absence of renal failure, identified risk factors are hypercoagulable states, malignancy, hyperparathyroidism, connective tissue disease, corticosteroids, vitamin D deficiency, calcium based phosphate binders, warfarin, obesity, and diabetes [1–5]. This patient's risk factors were gender, hyperparathyroidism, connective tissue disease, corticosteroid, and warfarin use. Although risk factors for NUCA have been identified the pathogenesis remains poorly understood.

In CUA chronic renal failure causes excess phosphate, which binds with calcium in the blood resulting in blood vessel damage. Reduced vitamin D levels result in increased PTH production. As bones become resistant to PTH the parathyroid glands enlarge to produce more PTH and calcium levels in the blood increase. Although the initial mechanism resulting in calcium deposition in NUCA is not as well characterised as CUA a final common pathway for both CUA and NUCA has been proposed. This pathway involves elevated nuclear factor kB, leading to vascular calcification and thrombosis [4]. Via this pathway, chronic inflammation, impaired liver function, estrogens, parathyroid hormone abnormalities, warfarin, and glucocorticoids may all contribute to the disease.

In this case, given the normal renal function, calcium, phosphate, and vitamin D levels, the elevated PTH represents tertiary hyperparathyroidism. The initial creatinine rise was attributed to hypovolemia caused by aggressive CCF management. Satellite purpuric ulcerations with central overlying necrosis crusts, present in this case, typify the clinical lesions of NUCA. Livedo reticularis or racemosa is usually present on the legs and may precede ulcerations. Clinicopathological correlation is essential for a diagnosis of NUCA.

The characteristic histological features of NUCA, as evident in this case, are calcification of small-medium sized cutaneous vessels, intimal fibrosis, intravascular thrombi, and diffuse calcification of small capillaries in the fat. There may be calcification of adipocytes. Epidermal and dermal ulceration give rise to the typical clinical features of NUCA. Investigations such as plain film X-rays or bone scintigraphy can show arteriolar calcification which may aid diagnosis or monitoring of treatment effects [6].

The management of NUCA involves elimination of identifiable precipitants, adequate analgesia, and early treatment. Meticulous wound management decreases infection risk, and surgical debridement may be warranted. Recent research has suggested that changing warfarin to low molecular weight heparin may be beneficial [5]. Rivaroxaban rather than low molecular weight heparin replaced warfarin in this case due to atrial fibrillation. Other reported treatments are calcimimetic agents, bisphosphonates, hyperbaric oxygen, low dose tissue plasminogen activator, and doxycycline [7]. There is limited evidence for intravenous sodium thiosulfate for its antioxidative properties and ability to increase calcium solubility in NUCA [8].

## 4. Conclusion

This rare case of NUCA in an 82-year-old Caucasian woman coincided with her hospitalization for the management of her CCF. Her risk factors were gender, connective tissue disease, hyperparathyroid level, glucocorticoid use, and warfarin use. Clinical, histological, and radiological evidence were consistent with a diagnosis of NUCA with no sinister precipitating factors found. The patient improved with meticulous wound care, analgesia, doxycycline 50 mg/day, Zoledronic acid 5 mg, and warfarin cessation. Given the rarity of the disease dermatologists and patients alike would benefit from a greater awareness of NUCA.

## Figures and Tables

**Table 1:** Blood results.

Test	02.08.2015	21.09.2015	Normal range
Haemoglobin	138	129	115–160 g/L
White cell count	7.0	7.4	4–11 × 10^9^
Platelets	251	220	150–400 × 10^9^
Neutrophils	5.26	6.02	1.8–7.5 × 10^9^
Urea	6.7	12.6	2.5–7.5 mmol/L
Creatinine	96	120	45–90 *μ*mol/L
GFR est	48	36	>90 Mls/min
Glucose	5.0	6.6	3.5–5.5 mmol/L
Osm-calc	291	290	280–300 mOsm/kg
Bilirubin total	25	23	2–20 *μ*mol/L
ALT	14	29	9–33 U/L
Alkaline phosphatase	101	140	30–110 U/L
New GGT	295	502	9–56 g/L
Protein	69	67	69–80 g/L
Albumin	41	43	33–50 g/L
ESR	32	43	1–40 mm/hr
CRP	7.3		<6 Mg/L
ACE level (Buhlmann)	49.8		20–70 U/L
Calcium		2.36	2.1–2.6 mmol/L
Corrected calcium		2.42	2.1–2.6 mmol/L
Phosphate		1.39	0.75–1.50 mmol/L
Intact serum PTH		17.7	1.6–7.2 pmol/L
25-OH vitamin D		82	<60 nmol/L
Urinary Creatinine		5.7	5.3–15.9 mmol/24 h
Urinary calcium		0.9	2.5–7.5 mmol/24 h
Cold agglutinin		Negative	
Cryoglobulin		Negative	
C3		1.58	0.75–1.61 g/L
C4		0.44 H	0.13–0.40 g/L
IgG		5.3	6.5–15.2 g/L
IgA		1.06	0.76–3.89 g/L
IgM		0.6	0.3–2.3 g/L
ANA speckled		1 : 80	Negative
ENA		Negative	<30 AU/mL
dsDNA		5	<40 IU/mL
ANCA		Atypical	Negative
MPO ANCA		Negative	Negative
PR3 ANCA		Negative	Negative
Globulins		Normal	Normal

## Abbreviation List

CUA: Calcific uremic arteriolopathy
CCF: Congestive cardiac failure
NUCA: Nonuremic calcific arteriolopathy
PTH: Parathyroid hormone.
